# ETV5 overexpression promotes progression of esophageal squamous cell carcinoma by upregulating SKA1 and TRPV2

**DOI:** 10.7150/ijms.71892

**Published:** 2022-06-21

**Authors:** Ming-Chuang Sun, Kang Fang, Zhao-Xing Li, Yuan Chu, Ai-Ping Xu, Zi-Ying Zhao, Zhu-Yun Leng, Yun-Wei Zhang, Ze-Hua Zhang, Li Zhang, Tao Chen, Mei-Dong Xu

**Affiliations:** 1 Endoscopy Center, Department of Gastroenterology, Shanghai East Hospital, School of Medicine, Tongji University, Shanghai, 200120, China; 2 Department of Pathology, Shanghai East Hospital, School of Medicine, Tongji University, Shanghai, 200120, China; Ming-Chuang Sun and Kang Fang contributed equally to this work.

**Keywords:** Esophageal squamous cell carcinoma, ETV5, SKA1, TRPV2, MMPs, metastasis

## Abstract

Esophageal squamous cell carcinoma (ESCC) is notorious for the rapid progression especially early tumor metastasis due to the unclear mechanism. Recently, ETV5 attracts much attention for its potential role as an oncogenic transcription factor involved in multiple cancers. However, no one reported the mechanism behind the association between ETV5 expression and esophageal squamous cell carcinoma progression. In this study, we found that ETV5 was upregulated in ESCC both from online database and our ESCC tissues and ETV5 was associated with tumor staging and prognosis. Knockdown of ETV5 or its downstream genes SKA1 and TRPV2 significantly suppress ESCC cells migration and invasion, respectively. Additionally, in vivo study showed knockdown of ETV5 inhibited tumor metastasis. Further experiments unveiled ETV5 could transcriptionally upregulate the expression of SKA1 and TRPV2 and further activate MMPs in ESCC progression. In conclusion, ETV5 was associated with ESCC tumor staging and ESCC prognosis clinically. ETV5 promoted metastasis of ESCC by activating MMPs through augmenting the transcription of SKA1 and TRPV2. ETV5 was likely to be a novel oncogene and therapeutic target in ESCC.

## Introduction

Esophageal cancer is a global health problem with an overall 5-year less than 20% 1. So far, China occupied the most significant proportion in esophageal cancer cases and deaths worldwide and esophageal squamous cell carcinoma (ESCC) was the main histological type 2, 3. ESCC is notorious for its rapid progression, especially early tumor metastasis and lacking effective treatment. Therefore, much attention should be paid to explore the biological behavior, diagnosis markers, and approach targets of ESCC.

As one of the largest families of signal-dependent transcription factors, the E26 transformation-specific (ETS) family comprises 28 homologs. The PEA3 subset of the ETS family, contains three factors: ETV1/ER81, ETV4/PEA3/E1AF, and ETV5/ERM 4. Recently, ETV5 has attracted much attention for its important role as an oncogenic transcription factor in multiple cancer types and its involvement in multiple biological processes. For example, ETV5 is related to EMT in papillary thyroid cancer 5. ETV5 accelerates tumor growth by promoting cell cycle G1/S transition in colorectal cancer 6. Additionally, ETV5 is linked to the maintenance of cancer stem cell (CSC) phenotype in breast cancer 7. Yet to date, the role of ETV5 in ESCC is still unknown. Through TCGA and GEO databases, we found ETV5 was significantly elevated in ESCC, but the true function of ETV5 in ESCC has never been studied. Thus, to evaluate and identify the role of ETV5 in ESCC development and progression, further study is needed.

In this study, we investigated the expression and function of ETV5 in ESCC and further identified its downstream targets by employing both in vitro and in vivo assays for the first time. The results revealed the oncogenic role of ETV5 in ESCC progression.

## Materials and Methods

### Data collection

The Cancer Genome Atlas (TCGA) is a large-scale cancer genomics program, and it has molecularly characterized 33 primary cancer types comprising esophageal squamous cell carcinoma. GEO is a public genomics platform composited of array- and sequence-based data. Using UALCAN and Oncomine, the expression of ETV5 in ESCC was investigated from TCGA and GEO database.

### Cell culture

We purchased human ESCC cell lines, ECA109, KYSE150 and TE1 from the Institute of Biochemistry and Cell Biology of the CAS (Shanghai, China). All cell lines were cultured in DMEM medium (GIBCO) supplemented with 10% FBS (GIBCO) in an incubator containing 5% CO_2_ at 37℃.

### Cell transfection

For transient knockdown transfection, cells were plated in 6-well plates and transfected with 100pmol siRNA-ETV5 or siRNA-SKA1 or siRNA-TRPV2 (GenePharma, Shanghai, China) each well. For transient gene overexpression transfection, ETV5 cDNA was synthesized and inserted into the pLVX plasmid to overexpress gene ETV5. Real-time quantitative polymerase chain reaction (RT-qPCR) and western blotting were applied to detect transfection efficiency at 48h after transfection. For stable transfection, lentivirus vectors that encode a shRNA targeting ETV5 or shRNA non-targeting control were used to transfect ECA109 cells following the manufacturer's instruction (Genechem, Shanghai, China). In brief, cells were plated in 6-well plates and medium containing viral fluid but without serum was added when the cell density reached 30% and replaced with complete medium 24h later. The transfection efficiency of each vector was detected via western blotting.

### Migration and invasion assay

To explore the migration ability, we resuspended ESCC cells in 200μl DMEM without serum and added them in upper chamber of the transwell device, with 5×10^4^ cells/well. We then added 600μl complete medium into the lower chamber as the chemical attractants. After incubation for 48h at 37℃, cells on the lower surface of the non-coated membrane were fixed by 4% paraformaldehyde and then stained by Giemsa. Images from five representative fields of each membrane were taken by using a light microscope (100×). The number of migratory cells was counted and the relative migration rate can be calculated. Invasion assay was similar to migration assay, but with the difference that 100μl of 200μg/ml diluted Matrigel matrix (Corning, 356234) was carefully added to the center of each transwell insert and incubated at 37℃ for 2 hours to form a gel before cells were plated.

### Wound healing assay

The ECA109 and KYSE150 cells underwent a culturing process in six-well plates and when the cell density reached 80%, the monolayers were scraped by the tip of a 200μl pipette, and cells continued to be cultured in DMEM free of serum. At 0, 24 and 48 h after scratch, cell migration was photographed by a light microscope (100×). Image J was used to calculate the closure. The formulas are: average scratch width = scratch gap area/length; the relative cell migration rate = (the scratch width at 0h-scratch width after culture)/the scratch width at 0 h × 100%.

### Real-time PCR assay and RNA sequencing

Total RNAs were extracted from cells by using Trizol solution, according to the instruction of the manufacturer. We used Nanodrop2000 spectrophotometer to measure the RNA concentrations and synthesized complementary DNA (cDNA) from RNA by using a PrimeScript RT reagent kit (Takara, Japan). TaqMan real-time PCR assays for ETV5, SKA1 and TRPV2 were applied following the instruction of Takara Bio. The relevant primers were summarized in supplementary table 1. All reactions, including the no-template controls were run in triplicate. After the reactions, the CT values were determined using fixed threshold settings. Data was analyzed using the 2^-△△CT^ method. Library preparation for RNA sequencing was conducted. Generally, 1 μg high-quality RNA was used, and sequencing was carried out by HiSeq2500 (Illumina Inc., San Diego, CA) at Genechem Biotechnology (Shanghai) Co., Ltd.

### Western blotting analysis

After being washed by PBS, all kinds of cell lines were harvested in RIPA buffer. The protein concentrations were measured using Bradford assay. Samples were separated on 8-12% SDS-PAGE gels and then transferred to PVDF membrane (Millipore, USA) and probed with primary antibodies specific for ETV5 (ab102010, Abcam), MMP2 (ab92536, Abcam), MMP9 (ab76003, Abcam), SKA1 (bs-7846R, Bioss) and TRPV2 (bs-10297R, Bioss). β-Actin, Tubulin and GAPDH were used as loading controls for western blots.

### Chromatin immunoprecipitation (CHIP) assay

A CHIP assay was performed using an Upstate Biotechnology kit. Briefly, we successively subjected cells to the procedures containing DNA-protein cross-linking, disruption of membrane and cytosol. Samples were digested by MNase and sonicated and then precipitated with antibody against transcriptional factor ETV5. Quantitative real-time PCR was used to measure the amount of bound DNA. According to the relative amount of input and the IgG ratio, the enrichment value was calculated. The primers covering ETV5 binding site of SKA1 and TRPV2 gene promoter region were summarized in supplementary table 1.

### Dual luciferase reporter assay

ESCC cells were seeded into 12-well plates at a density of 2.5×10^4^ cells/well. The reporter plasmid containing wild SKA1 or TRPV2 promoter and mutant SKA1 or TRPV2 promoter at the binding site, was transfected into cells, respectively. The cells were also transfected with Renilla luciferase reporter plasmids for signal normalization. After 24h, the luciferase activity was measured by the Dual Luciferase Assay System Kit (Promega, Madison, WI, USA).

### Immunohistochemistry (IHC)

This study was approved by the institutional review board of Shanghai East Hospital, Tongji University. ESCC and corresponding healthy esophageal mucosa (CHEM) tissues of 79 patients were collected. No patient received previous treatment, such as surgery, chemotherapy, and radiotherapy. The details of Clinicopathological factors were shown in supplementary table 2. After being fixed by Formalin and embedded by paraffin, tissue sections were cut to 4μm thickness and then placed in xylene and graded alcohols for deparaffinization and hydration. We performed heat-induced antigen retrieval in EDTA (PH 8.0) buffer for 15 minutes by using a microwave oven. To reduce nonspecific staining, we performed blocking with 10% goat serum. After the specific primary anti-ETV5 (ab102010, Abcam) was dropped onto the sections and incubated overnight at 4℃, the slides were counterstained with light hematoxylin, dehydrated, and cover-slipped.

### Animal studies

This in vivo study was approved by the animal care and use committee of Tongji University. 16 female BALB/c nude mice (6 weeks old) were used for animal studies. The animals were randomly divided into 2 groups (control and treated groups, 8 mice per group). ECA109 cells were treated with stable transfection. After cell harvest, cells were resuspended and injected into the tail vein of each mouse (2×10^6^ viable ECA109 cells/mouse). At 6 weeks, the lung metastasis was monitored. According to the AVMA Guidelines for the Euthanasia of Animals, we performed intraperitoneal injection of a three-fold dose of barbiturates to euthanize all the mice. After that, lungs were removed, and the lung colonization number was counted. Serial sections of lung tissue were stained with hematoxylin and eosin.

### Statistical analysis

The statistical analysis was performed by SPSS 19.0 (IBM, Armonk, NY, USA). All the experiments were carried out repeatedly three times. Differences between groups were calculated using Student's t-test, Chi-square test, or Fisher's exact test. The p value<0.05 was considered with statistical significance. Kaplan Mier methods and log-rank test were used to analyze survival data.

## Results

### ETV5 expression is upregulated in esophageal squamous cell carcinoma

**Figure 1:**
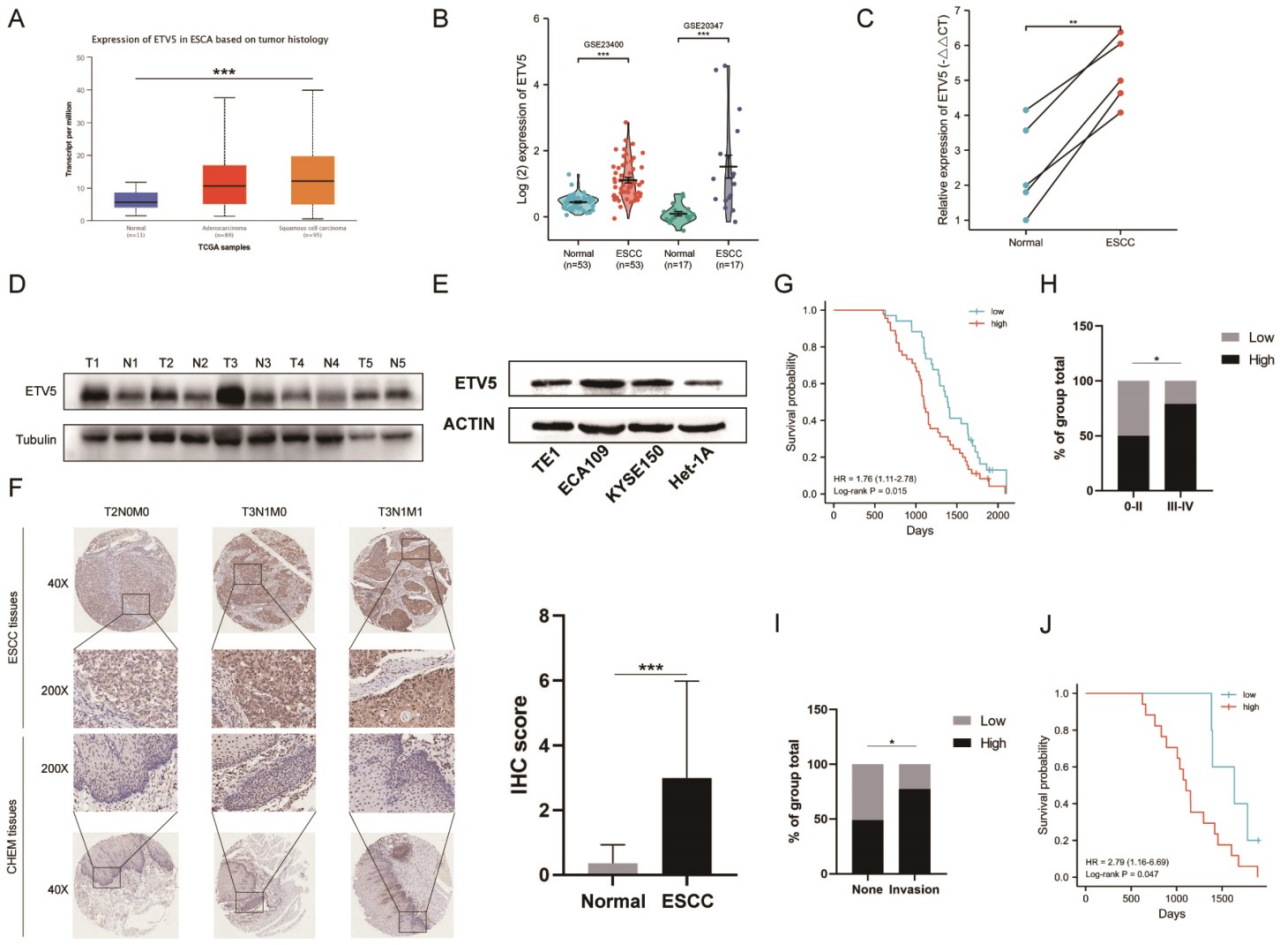
ETV5 is upregulated and correlated to tumor staging and prognosis in escc. The level of ETV5 mRNA is upregulated in ESCC tissues indicated by data from TCGA(A) and GEO(B). (C)(D) RT-PCR and western blotting analysis indicated that the expression level of ETV5 was higher in selected ESCC tissues than in paired normal esophageal mucosa tissues. (E) Western blotting analysis showed that ETV5 was elevated in ESCC cell lines. (F) IHC analysis showed that the expression level of ETV5 was higher in our ESCC tissues. (G) Kaplan-Meier analysis indicated high expression level of ETV5 is associated with reduced overall survival in our clinical tissue samples. (H) ETV5 protein level was higher in Stage III-IV ESCC. (I) ETV5 protein level was higher in invasion group. (J) Kaplan-Meier analysis indicated high expression level of ETV5 is associated with reduced overall survival in invasion group. *P<0.05; **P<0.01; ***P<0.001.

To investigate the expression of ETV5 in ESCC, we initially analyzed the online data from TCGA and GEO by using UALCAN and Oncomine, respectively. ETV5 is significantly upregulated in ESCC, not only supported by the RNA-sequencing data from TCGA (Figure 1A), but also validated by data from GEO (Figure 1B). This result was further confirmed after testing ETV5 expression in ESCC tissues and paired normal esophageal mucosa tissues in 5 randomly selected ESCC patients. RT-PCR and western blotting assays showed significantly higher expression of ETV5 in ESCC tissues than in the normal esophageal mucosa tissues, both at the mRNA and protein levels (Figure 1C, D). In the aspect of cell lines, the expression of ETV5 is higher in ESCC cells than that in normal esophageal epithelial cells (Figure 1E). Besides, IHC analysis also showed that upregulation of ETV5 was found in our ESCC tissues (Figure 1F).

### Elevated ETV5 protein expression is associated with poor prognosis of ESCCs

Kaplan-Meier analysis in our study indicated that patients whose samples have high ETV5 expression level have significantly shorter progression free survival and overall survival (Figure 1G). Furthermore, by analyzing the correlation of clinicopathologic parameters with ETV5 level in ESCC tissues, we found that ETV5 protein level was higher in Stage III-IV cancers than in Stage 0-II cancers (Figure 1H). To pay more attention to the correlation between ESCC invasion especially metastasis and ETV5 protein level, we defined metastasis in lymph node or in other organs as invasion group, none metastasis as none group. We found that ETV5 protein level was higher in invasion group than in none group (Figure 1I). What's more, in invasion group, patients whose samples have high ETV5 expression level have significantly shorter progression free survival and overall survival (Figure 1J).

### ETV5 silencing suppresses migration and invasion of ESCC cells

**Figure 2:**
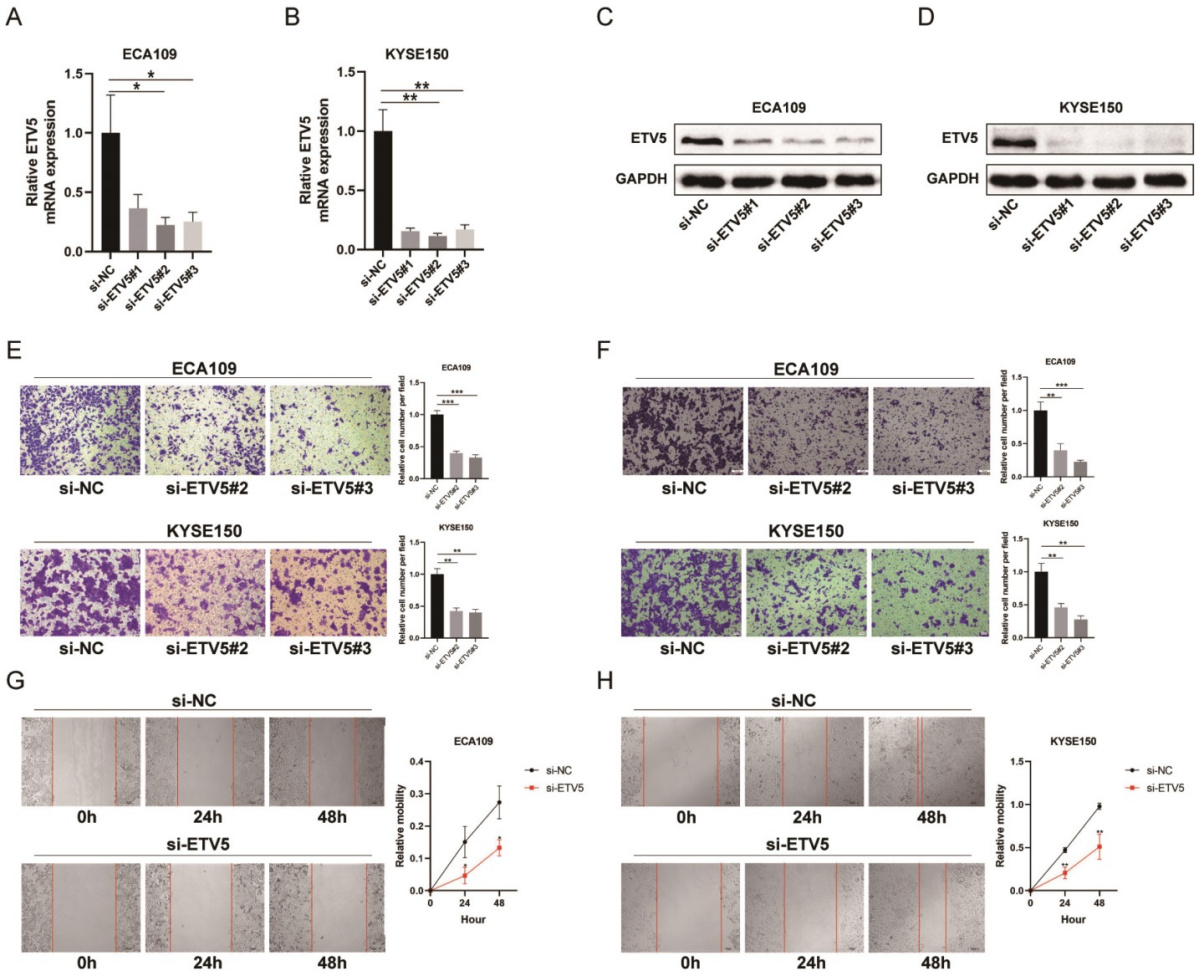
ETV5 affects cell migration and invasion in ESCC. (A)(B) QPCR indicated that ETV5 was significantly inhibited by siETV5 in ECA109 and KYSE150 cells. (C)(D) Western blot indicated that ETV5 was significantly inhibited by siETV5 in ECA109 and KYSE150 cells. (E) ETV5 knockdown markedly inhibited migration of ECA109 and KYSE150 cells indicated by migration assay. (F) ETV5 knockdown markedly inhibited invasion of ECA109 and KYSE150 cells indicated by invasion assay. (G)(H) ETV5 knockdown markedly suppressed migration of ECA109 and KYSE150 cells, as determined via wound healing assay. *P<0.05; **P<0.01; ***P<0.001.

To further study how ETV5 affects the functions of ESCC cells, migration, invasion and wound healing assays were performed. The expression of ETV5 was knocked down by transfecting specific siRNA targeting ETV5, indicated by real-time PCR (Figure 2A, B) and western blot results (Figure 2C, D). The results of the migration (Figure 2E) and wound healing assays (Figure 2G, H) indicated that cell migration in these two cell lines was obviously suppressed when ETV5 was knocked down. Additionally, the results of invasion assay also showed that knockdown of ETV5 evidently decreased cell invasion in these two cell lines (Figure 2F).

### ETV5 transcriptionally regulates SKA1 and TRPV2

**Figure 3:**
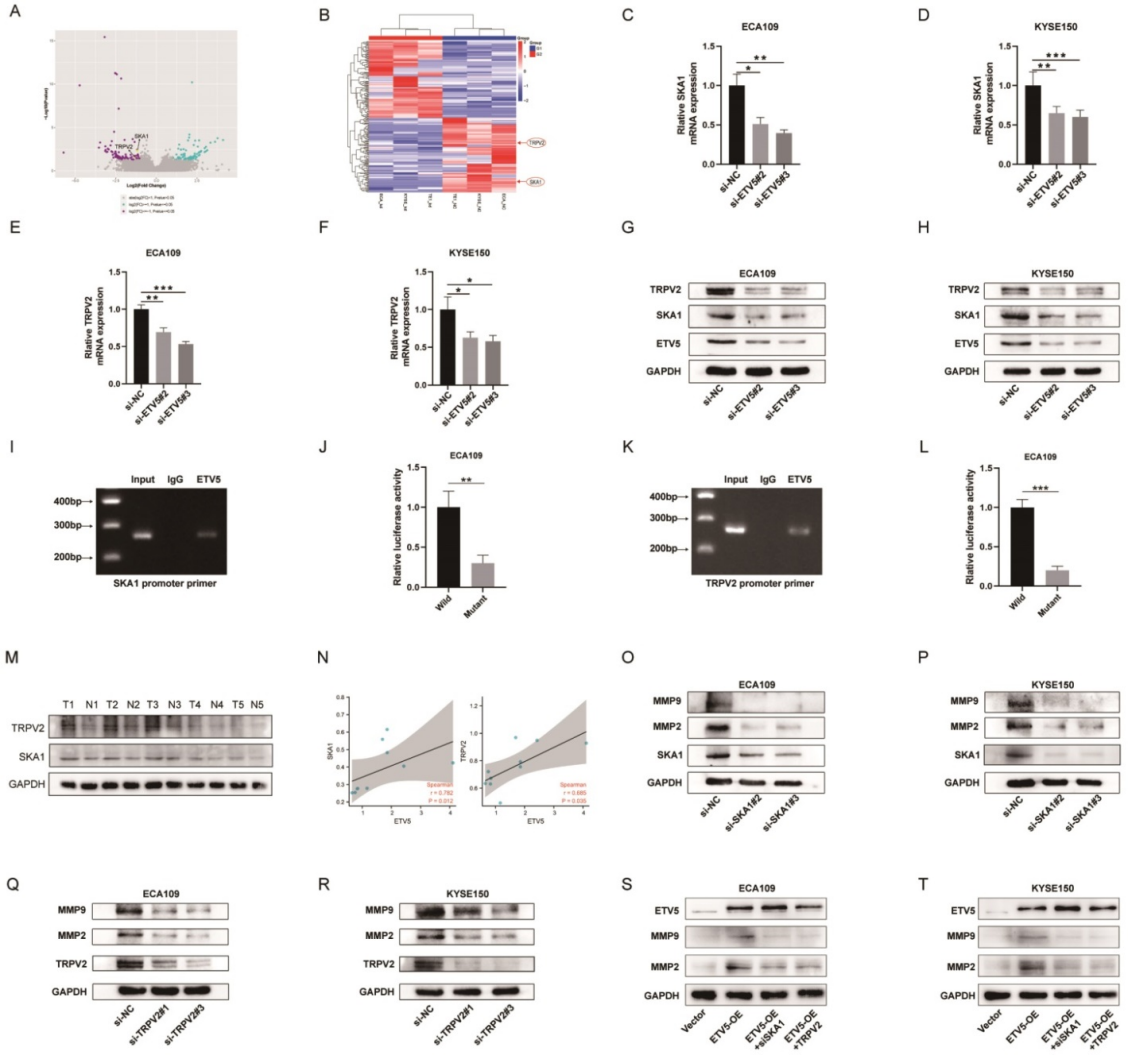
ETV5 activates MMPs through regulating SKA1 and further regulating TRPV2 in ESCC. (A)(B) Volcano Plot and Clustering analysis showed significantly upregulated and downregulated gene signature in ECA109, KYSE150, or TE1 siETV5 groups normalized to NC siRNA groups by RNA-seq. (C-F) QPCR indicated that the knockdown of ETV5 could downregulate SKA1 and TRPV2 mRNA expression in ECA109 and KYSE150 cells. (G)(H) Western blot showed that knockdown of ETV5 could downregulate SKA1 and TRPV2 protein expression in ECA109 and KYSE150 cells. (I) Chip assay revealed that a positive signal was detected via primers targeting SKA1. (J) Luciferase reporter gene assays showed that mutant at the predicted binding site of SKA1 significantly decreased the luciferase activity in ECA109 cells. (K) Chip assay revealed that a positive signal was detected via primers targeting TRPV2. (L) Luciferase reporter gene assays showed that mutant at the predicted binding site of TRPV2 significantly decreased the luciferase activity in ECA109 cells. (M)Western blot indicated that protein SKA1 and TRPV2 were higher in selected ESCC tissues than in paired normal esophageal mucosa tissues. (N) Correlation analysis indicated that ETV5 was in positive correlation with SKA1 and TRPV2 respectively. (O)(P) Western blot indicated that SKA1 knockdown markedly downregulated protein expression of MMPs in EAC109 and KYSE150 cells. (Q)(R) Western blot indicated that TRPV2 knockdown markedly downregulated protein expression of MMPs in ECA109 and KYSE150 cells. (S)(T) Western blot indicated that SKA1 and TRPV2 could significantly rescue the effects of ETV5 overexpression on MMPs in ECA109 and KYSE150 cells. *P<0.05; **P<0.01; ***P<0.001.

In order to identify the molecular mechanism underlying the ETV5-mediated increase in cancer progression, RNA-seq analysis was performed to compare protein-coding transcripts levels in ECA109, KYSE150 and TE1 cells treated with or without siETV5. With P<0.05 and FC>2 as cutoff values, 100 downregulated genes in siETV5 transfected cells were found (Figure 3A, B). Among the 100 genes, SKA1 and TRPV2 attracted our attention because though the mechanism was unclear, its' over-expression in ESCC had been reported^8^, ^9^. Additionally, it has been elucidated that SKA1 or TRPV2 could promote tumor progression via augmenting cell migration and invasion abilities in some other cancers^10^, ^11^. We performed real-time PCR and western blot validation in ESCC cells after knockdown ETV5. In ECA109 and KYSE150 cells transfected with siETV5, SKA1 and TRPV2 mRNA were significantly downregulated (Figure 3C-H). Considering ETV5 is a transcription factor, we proposed that ETV5 may exert its functions by regulating the transcription of SKA1 and TRPV2. CHIP assay was done to verify the binding site of ETV5 in the SKA1 and TRPV2 (Figure 3I, J). Besides, luciferase assays demonstrated that ETV5 could increase SKA1 and TRPV2 transcriptional activation (Figure 3K, L). In the tissues of the same 5 ESCC patients, the expression level of protein SKA1 and protein TRPV2 were also significantly higher in ESCC tissues than in corresponding normal tissues (Figure 3M). Furthermore, through correlation analysis, we found that they are in positive correlation with protein ETV5 respectively (Figure 3N). Moreover, MMP2 and MMP9, which were reported to be downstream of SKA1 or TRPV2 in many other cancers, were found to be downregulated in ESCC cells when SKA1 or TRPV2 was knocked down by siRNAs (Figure 3M-P). From the results of rescue assay, we found that when ETV5 was overexpressed, whatever SKA1 knockdown or TRPV2 knockdown could obviously rescue the effects of ETV5 on MMPs (Figure 3S, T).

### SKA1 or TRPV2 silencing suppresses migration and invasion of ESCC cells

**Figure 4:**
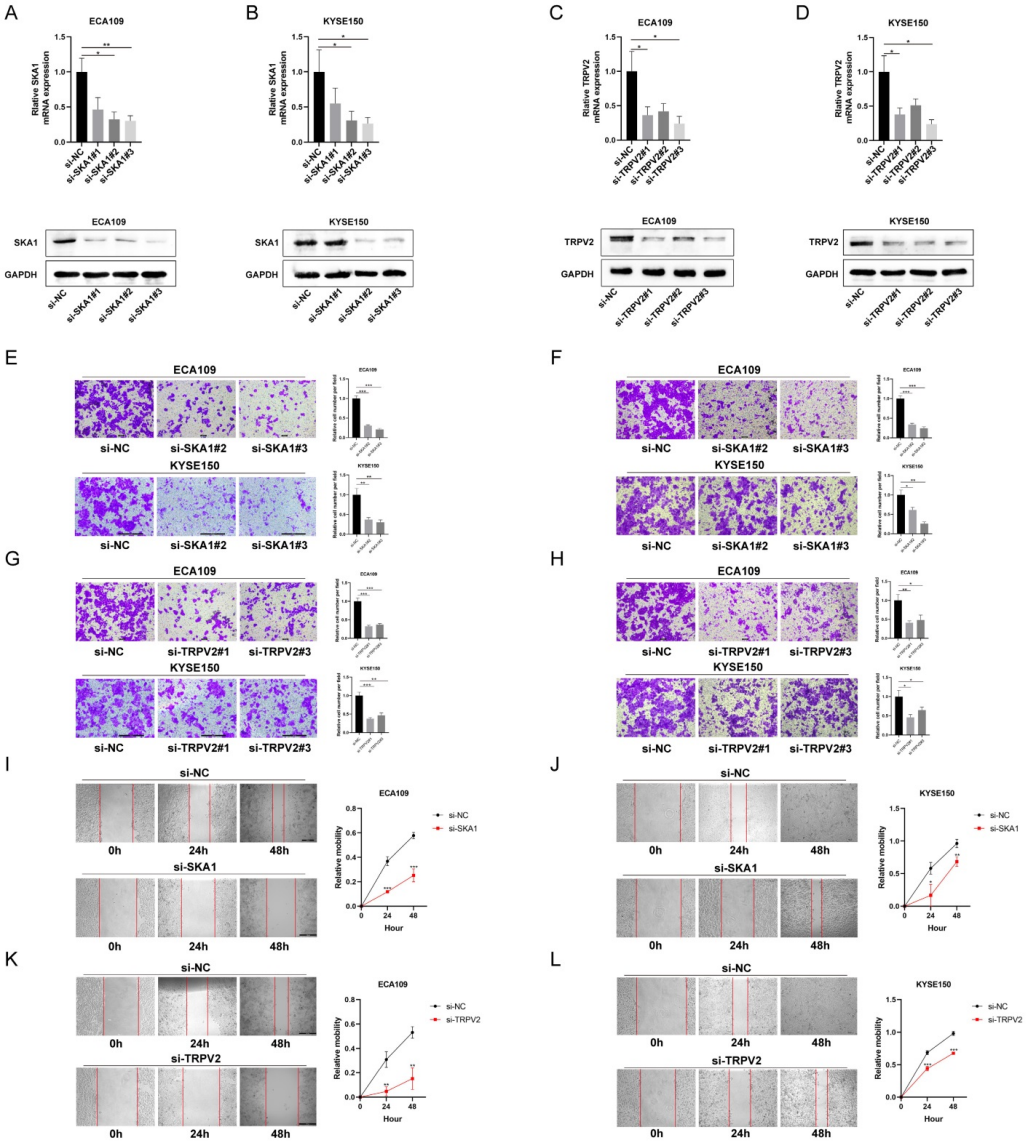
SKA1 and TRPV2 affected cell migration and invasion in ESCC. (A)(B) QPCR and western blot indicated that SKA1 was significantly inhibited by siETV5 in ECA109 and KYSE150 cells. (C)(D) QPCR and western blot indicated that TRPV2 was significantly inhibited by siETV5 in ECA109 and KYSE150 cells. (E) SKA1 knockdown markedly inhibited migration of ECA109 and KYSE150 cells indicated by migration assay. (F) SKA1 knockdown markedly inhibited invasion of ECA109 and KYSE150 cells indicated by invasion assay. (G) TRPV2 knockdown markedly inhibited migration of ECA109 and KYSE150 cells indicated by migration assay. (H) TRPV2 knockdown markedly inhibited invasion of ECA109 and KYSE150 cells indicated by invasion assay. (I)(J) SKA1 knockdown markedly suppressed migration of ECA109 and KYSE150 cells, as determined via wound healing assay. (K)(L) TRPV2 knockdown markedly suppressed migration of ECA109 and KYSE150 cells, as determined via wound healing assay. *P<0.05; **P<0.01; ***P<0.001.

To explore the function of SKA1 and TRPV2 in ESCC cells, migration, invasion and wound healing assays were performed. The expressions of SKA1 and TRPV2 were knocked down by transfecting specific siRNA, respectively, indicated by real-time PCR and western blot results (Figure 4A-D). The migration and wound healing assays results indicated that cell migration in these two cell lines was suppressed when whatever SKA1 or TRPV2 was knocked down (Figure 4E-L). It could also be easily detected that cell invasion in these two cell lines was decreased when whatever SKA1 or TRPV2 was knocked down from the invasion assays (Figure 4F, H).

### Effect of ETV5 on the in vivo metastasis of ECA109

**Figure 5:**
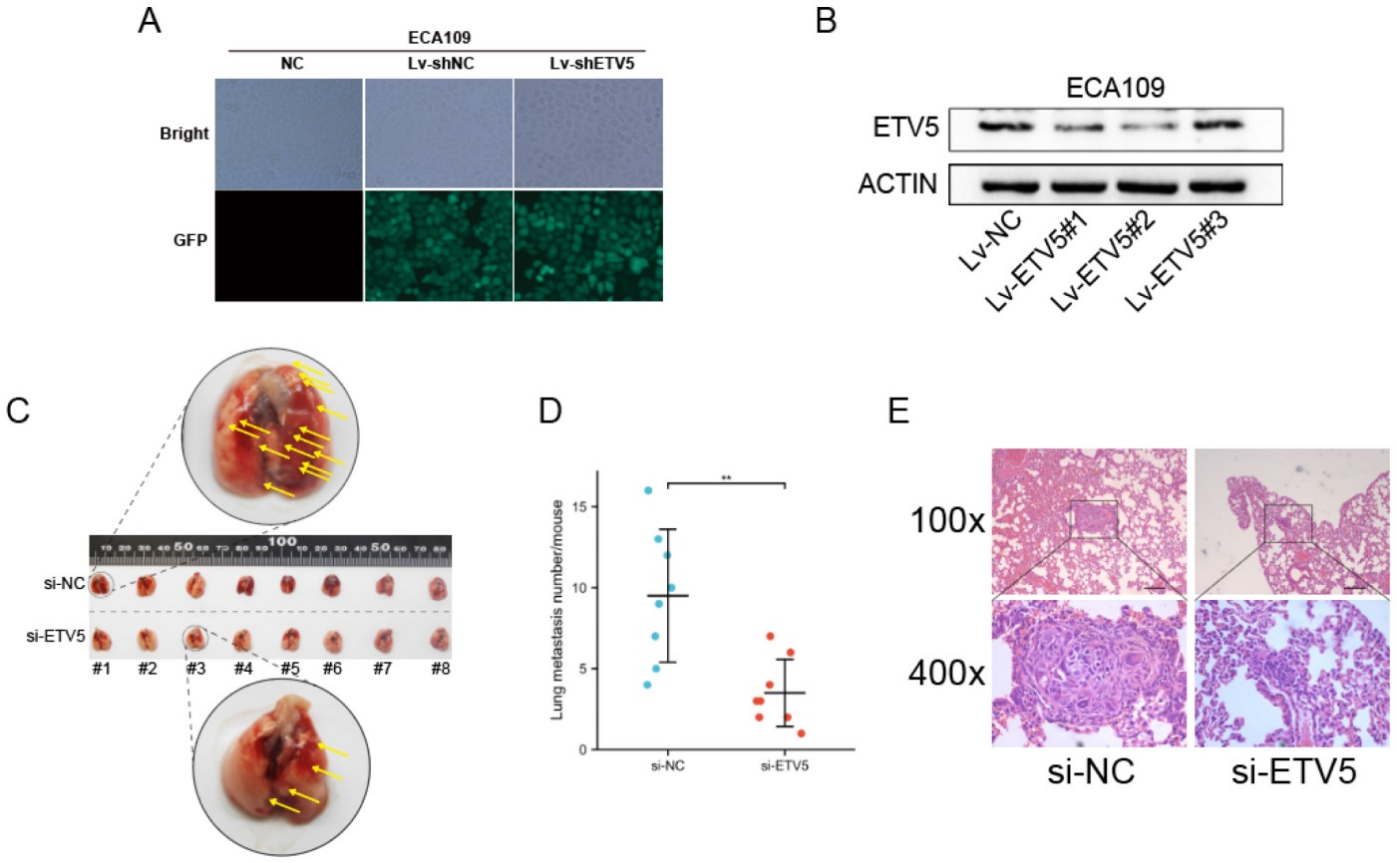
ETV5 affects ESCC metastasis in vivo. (A) Four days after lentivirus infection, more than 90% of ECA109 cells were GFR positive in both Lv-shRNA and Lv-shETV5 groups, as shown by fluorescence microscopy. (B) Western blot indicated that ETV5 was significantly inhibited by Lv-shETV5 in ECA109 cells. (C-E) Effects of ETV5 knockdown on colonization in the mice lungs after tail vein injection of ECA109-derived cells, including shETV5 or negative control group. Lung images were performed to detect metastasis foci (C). Metastasis quantification was evaluated (D). Representative hematoxylin and eosin (H&E) staining of lung sections (E). *P<0.05; **P<0.01; ***P<0.001. - represents 100 μm.

We further studied the effect of ETV5 on cancer metastasis by mice modes. High transfection and knockdown efficacy of lentivirus vectors that encode a shRNA targeting ETV5 were validated firstly (Figure 5A, B), and Lv-ETV5#2 was chosen to perform the following in vivo assay. In the tail vein injection nude mouse model, the average number of lung metastasis per mouse was significantly reduced in shETV5-transfected ECA109 cells compared with the control cells (Figure 5C, D). Histological analysis demonstrated that the metastatic nodules formed by shETV5-transfected cells were smaller than those formed by controlled cells (Figure 5E).

## Discussion

ESCC always has a poor prognosis because of its inconspicuous symptoms and metastasis in the early stage, which limits effective therapies to a great extent. Thus, understanding its distinct biological process and find novel diagnostic markers and therapeutic targets is imperative. In this study, we found that the expression level of ETV5 was higher in ESCC than that in the normal esophagus, both from tissue and cell aspects, which was validated by analyzing the data from TCGA and GEO database. Furthermore, the expression level of ETV5 was not only correlated to tumor stage and ESCC metastasis from the IHC results, but also correlated to overall survival. These results demonstrated that ETV5 might act as an oncogene similar to ETS-1 12 and ETV4 13 in ESCC. We further applied in vitro and in vivo studies and found that knockdown of ETV5 significantly suppressed ESCC migration and invasion, which confirmed that ETV5 could augment ESCC metastasis.

Considering ETV5 is a transcription factor, ETV5 should regulate some downstream targets during ESCC progression. We further exerted RNA-seq analysis and found two interesting genes SKA1 and TRPV2. Though they were ever reported to be overexpressed in ESCC, the concrete function and underlying mechanism are still obscure. Kinetochore-associated complex includes SKA1, SKA2 and SKA3. This complex plays a leading role in stabilizing the attachment of spindle microtubules to kinetochores and maintaining the metaphase plate during mitosis 14, 15. Acting as an oncogene in many cancer types, SKA1 contributes to multiple biological behaviors, including cell circle, EMT, and Wnt/β-catenin pathways 10, 16. Thus, its proper function in ESCC is worth exploring. Transient receptor potential Vanilloid family is a non-selective calcium-permeable channel and acts as a cellular sensor for heat, stretch and osmosis 17. Recently, it has raised much attention that TRPV2 acts as a cancer biomarker and potential therapeutic target for many cancer types 18, but the specific function in ESCC is not very clear. Herein, we speculated that SKA1 and TRPV2 could promote ESCC progression, transcriptionally regulated by ETV5. Firstly, the results of in vitro studies verified the oncogenic roles of SKA1 and TRPV2 in migration and invasion of ESCC cells, so they could also augment ESCC metastasis. Additionally, the results of Real-time PCR and western blotting analysis indicated ETV5 could regulate the expression of SKA1 and TRPV2. Moreover, the CHIP assays and luciferase assays results further told us that ETV5 could directly bind to the promotor of SKA1 and TRPV2 and activate their transcription.

As mentioned above, ETV5 could promote ESCC metastasis by directly regulating SKA1 and TRPV2, but the involved biological process was not clear. MMPs are crucial agents responsible for extracellular matrix degradation, and they are always considered as important factors accounting for tumor metastasis. Besides, they are common downstream factors of ETS protein, especially the PEA3 subfamily 19. Of MMPs, MMP2 and MMP9 were the main members presented to be regulated by SKA1 and TRPV2 in some cancers, such as human adenoid cystic carcinoma, non-small cell lung cancer and prostate cancer 20-22. Thus, these two MMPs may also take part in ESCC metastasis. Through western blotting analysis, we found the expression level of MMP2 and MMP9 are in accordance with that of SKA1 and TRPV2, which indicated both SKA1 and TRPV2 could promote ESCC metastasis by co-targeting MMP2 and MMP9. In this study, we demonstrated that ETV5 was overexpressed in ESCC cells, and correlated to poor prognosis. ETV5 promoted metastasis of ESCC by activating MMPs through augmenting the transcription of SKA1 and TRPV2. These findings provide new perspectives for the treatment of ESCC.

## Conclusion

This study described, for the first time, that ETV5 expression was significantly increased in ESCC tissues and it was associated with ESCC tumor staging and ESCC prognosis clinically. Additionally, ETV5 promoted the progression of ESCC and ETV5 activated MMPs through transcriptionally regulating SKA1 and TRPV2 in ESCC. ETV5 was likely to be a novel oncogene and therapeutic target in ESCC.

## Supplementary Material

Supplementary tables.

## References

[1] Thrift AP (2021). Global burden and epidemiology of Barrett oesophagus and oesophageal cancer. Nature reviews Gastroenterology & hepatology.

[2] Kamangar F, Nasrollahzadeh D, Safiri S, Sepanlou S (2020). The global, regional, and national burden of oesophageal cancer and its attributable risk factors in 195 countries and territories, 1990-2017: a systematic analysis for the Global Burden of Disease Study 2017. The lancet Gastroenterology & hepatology.

[3] Arnold M, Soerjomataram I, Ferlay J, Forman D (2015). Global incidence of oesophageal cancer by histological subtype in 2012. Gut.

[4] Qi T, Qu Q, Li G, Wang J, Zhu H, Yang Z (2020). Function and regulation of the PEA3 subfamily of ETS transcription factors in cancer. American journal of cancer research.

[5] Puli OR, Danysh BP, McBeath E, Sinha DK, Hoang NM, Powell RT (2018). The Transcription Factor ETV5 Mediates BRAFV600E-Induced Proliferation and TWIST1 Expression in Papillary Thyroid Cancer Cells. Neoplasia (New York, NY).

[6] Peng Y, Feng H, Wang C, Song Z, Zhang Y, Liu K (2021). The role of E26 transformation-specific variant transcription factor 5 in colorectal cancer cell proliferation and cell cycle progression. Cell death & disease.

[7] Yoe J, Kim D, Kim S, Lee Y (2020). Capicua restricts cancer stem cell-like properties in breast cancer cells. Oncogene.

[8] Hu D, Li Z, Li X, Fu H, Zhang M (2019). SKA1 overexpression is associated with the prognosis of esophageal squamous cell carcinoma and regulates cell proliferation and migration. International journal of molecular medicine.

[9] Zhou K, Zhang SS, Yan Y, Zhao S (2014). Overexpression of transient receptor potential vanilloid 2 is associated with poor prognosis in patients with esophageal squamous cell carcinoma. Medical oncology (Northwood, London, England).

[10] Li T, Liu X, Xu B, Wu W, Zang Y, Li J (2020). SKA1 regulates actin cytoskeleton remodelling via activating Cdc42 and influences the migration of pancreatic ductal adenocarcinoma cells. Cell Prolif.

[11] Kato S, Shiozaki A, Kudou M, Shimizu H, Kosuga T, Ohashi T (2021). TRPV2 Promotes Cell Migration and Invasion in Gastric Cancer via the Transforming Growth Factor-β Signaling Pathway. Annals of surgical oncology.

[12] He C, Wu S, Gao A, Su Y, Min H, Shang ZF (2017). Phosphorylation of ETS-1 is a critical event in DNA polymerase iota-induced invasion and metastasis of esophageal squamous cell carcinoma. Cancer science.

[13] Fung TM, Ng KY, Tong M, Chen JN, Chai S, Chan KT (2016). Neuropilin-2 promotes tumourigenicity and metastasis in oesophageal squamous cell carcinoma through ERK-MAPK-ETV4-MMP-E-cadherin deregulation. The Journal of pathology.

[14] Rahi A, Chakraborty M, Vosberg K, Varma D (2020). Kinetochore-microtubule coupling mechanisms mediated by the Ska1 complex and Cdt1. Essays in biochemistry.

[15] Huis In 't Veld PJ, Volkov VA, Stender ID, Musacchio A, Dogterom M (2019). Molecular determinants of the Ska-Ndc80 interaction and their influence on microtubule tracking and force-coupling. eLife.

[16] Wang X, Zeng Y, Zhou M, Zhang X, Xu A, Lin J (2019). SKA1 promotes malignant phenotype and progression of glioma via multiple signaling pathways. Cancer cell international.

[17] Clapham DE (2003). TRP channels as cellular sensors. Nature.

[18] Siveen KS, Nizamuddin PB, Uddin S, Al-Thani M, Frenneaux MP, Janahi IA (2020). TRPV2: A Cancer Biomarker and Potential Therapeutic Target. Disease markers.

[19] Seth A, Watson DK (2005). ETS transcription factors and their emerging roles in human cancer. European journal of cancer (Oxford, England: 1990).

[20] Zhao LJ, Yang HL, Li KY, Gao YH, Dong K, Liu ZH (2017). Knockdown of SKA1 gene inhibits cell proliferation and metastasis in human adenoid cystic carcinoma. Biomedicine & pharmacotherapy = Biomedecine & pharmacotherapie.

[21] Shen L, Yang M, Lin Q, Zhang Z, Miao C, Zhu B (2016). SKA1 regulates the metastasis and cisplatin resistance of non-small cell lung cancer. Oncology reports.

[22] Monet M, Lehen'kyi V, Gackiere F, Firlej V, Vandenberghe M, Roudbaraki M (2010). Role of cationic channel TRPV2 in promoting prostate cancer migration and progression to androgen resistance. Cancer research.

