# Supplementary material for: ETV5 overexpression promotes progression of esophageal squamous cell carcinoma by upregulating SKA1 and TRPV2
Source: Int J Med Sci. 2022 Jun 21;19(6):1072–81. doi: 10.7150/ijms.71892 (PMC9254378; doi:10.7150/ijms.71892)
Supplement: Supplementary file 1 — Supplementary tables. [file ijmsv19p1072s1.pdf]

Supplementary table 1 Primers for real-time PCR assays

ETV5	forward	5'-CAGCACACGGGTTCCAGTCAC-3'
	reverse	5'-TGGCAGTTAGGCACTTCTGAATCG-3'
SKA1	forward	5'-AGAGGGGAGAGGGGCTGGAG-3'
	reverse	5'-GCGTCACCTTCCTGGCACATC-3'
TRPV2	forward	5'-GATCGGCTCTTCAATGCGGTCTC-3'
	reverse	5'-CGGTGAGGTACTTGCTGGTCTTG-3'
SKA1(for CHIP)	forward	5'-AGGAAAGGACCCAGGCTTATCG-3'
	reverse	5'-ACCGTACTGACTTGTCTTAGAGCG-3'
TRPV2(for CHIP)	forward	5'-GGGCTGTCCACATCAGCTTTCTCC-3'
	reverse	5'-CAGGCTCCACCCCCACCC-3'

Supplementary table 2 The relationship between clinical factors and ETV5 expression

Clinicopathological factors	ETV5 expression		
	High	Low	P-value
**Gender**			
Male	36	29	0.542
Female	9	5	
**Age(y)**			
≤60	12	14	0.174
> 60	33	20	
**Tumor size**			
≤3cm	15	15	1
> 3cm	30	30	
**Metastasis***			
Negtive	28	29	0.024
Positive	17	5	
**TNM stage**			
0-II	30	30	0.026
III-IV	15	4	

* Metastasis here includes lymph node metastasis and other organs metastasis.
